# The efficacy of tourniquet assisted total knee arthroplasty on patient-reported and performance-based physical function: a randomized controlled trial protocol

**DOI:** 10.1186/1471-2474-15-110

**Published:** 2014-03-29

**Authors:** Rasmus Lohmann-Jensen, Anders Holsgaard-Larsen, Claus Emmeluth, Søren Overgaard, Carsten Jensen

**Affiliations:** 1 Orthopaedic Research Unit, Department of Orthopedic Surgery and Traumatology, Odense University Hospital, Odense C, Denmark; 2 Institute of Clinical Research, University of Southern Denmark, Odense C, Denmark

**Keywords:** Tourniquet, Total knee arthroplasty, Patient-reported, Performance-based, Physical function

## Abstract

**Background:**

Surgical treatment of osteoarthritis with total knee arthroplasty (TKA) usually takes place in a complete bloodless field using a tourniquet. However, doing the surgery without a tourniquet may reduce muscle damage, post-surgery pain and led to improved functional rehabilitation and mobilization.

**Methods/Design:**

A prospective, blinded, parallel-group, controlled superiority trial, with balanced randomization [1:1]. Patients aged 50 or older eligible for primary TKA for osteoarthritis will be consecutively recruited from Department of Orthopedic Surgery and Traumatology, Odense University Hospital, Denmark. A total of 80 patients will be randomly allocated to TKA with or without tourniquet application providing 40 patients for each of the two treatment arms. The tourniquet assisted TKA group will have an automatic, micro-processor-based pneumatic tourniquet inflated around the thigh during surgery. The non-tourniquet assisted TKA group will have surgery performed without application of a tourniquet. The primary aim is to compare tourniquet assisted to non-tourniquet assisted TKA on patient-reported physical function (KOOS-ADL). The secondary aim is to compare post-surgery pain, function in sports and recreation, quality of life, and performance-based physical function. The explorative outcomes include; use of pain medication, single-fiber muscle damage, and changes in mechanical muscle function. The primary endpoint will be at 3-months following surgical treatment, and the time-point for analysis of the primary outcome. However, follow-up will continue up to 1 year, and provide medium-term results. The treatment effect (difference in KOOS-ADL) will be analyzed using a random effects regression model, crude and adjusted results will be reported, if needed. Analyses will be based on the intention-to-treat (ITT). Subsequent per-protocol analysis may be necessary in the event of a substantial number of patients (> 15%) being lost during follow-up. The number needed to treat (NNT) for a positive effect of treatment (>10 points on KOOS-ADL) will be reported.

**Discussion:**

This is the first randomized clinical trial comparing the efficacy of tourniquet assisted TKA on patient-reported physical function supported by a range of performance-based secondary outcome measures. As such it will provide high quality evidence that may help determine whether tourniquet should be used in future TKA procedures in patients with osteoarthritis of the knee.

**Trial registration:**

ClinicalTrials NCT01891266.

## Background

Osteoarthritis (OA) is the most common joint disease and a significant cause of morbidity in middle-aged and older populations [1-3]. Knee OA is characterized by joint pain, swelling, reduced range of motion (ROM), decreased physical activity, and impaired quality of life [4]. Following the development of OA, the associated pain and disability generally increase with time [5,6]. In end-stage knee OA, total knee arthroplasty (TKA) may be the only effective treatment available to reduce pain and restore joint mobility and function.

TKA is an invasive procedure during which damaged cartilage is removed, joint deformities are corrected and the original knee joint is replaced with an artificial joint. The recovery period typically lasts for several months and patients often experience significant pain which may be only partly relieved by analgesia. Several factors such as the post-operative inflammatory response, muscle damage, pre-existing muscle weakness and swelling may contribute to the extensive recovery period. These factors may also negatively affect physical performance in the early post-surgery period.

Tourniquets inflated above systolic blood pressure have been used by surgeons for lower limb surgery for more than a century [7-10] and are still the preferred method in elective TKA [9,11-13]. The rationale for tourniquet use in TKA is primarily the optimization of intra-operative visibility and reduced blood loss [9,12,14,15]. However, such benefits must be weighed against the potential complications which include; increased risk of direct vascular injury [13,16], nerve palsy [17-19], deep-vein thrombosis [7,8,13,20] and subsequent pulmonary embolism [7,21,22]. Additionally, acute pulmonary edema and cardiac arrest immediately following tourniquet release have been reported [7,22,23]. There are also studies in the literature demonstrating that tourniquet use in TKA does not improve fixation [24,25]. Tourniquet use may also affect a patients’ physical recovery since its use causes microscopic muscle damage, post-surgery swelling, pain, and reduced knee joint ROM [7,24-28] and a greater understanding of patient-reported outcomes following TKA using a tourniquet is required. Furthermore, tourniquet use has been reported to cause post-surgery surface electromyography changes in the quadriceps muscles [29], and has been associated with increased levels of myoglobin in plasma [30], which collectively suggests pressure induced muscle damage does occur. Finally, patients undergoing non-tourniquet assisted TKA commenced rehabilitation exercises earlier and this may improve a patient confidence in using the new joint, decrease the incidence of complications associated with immobility and improve their overall satisfaction [7,29]. The use of tourniquet for TKA is therefore controversial and well-designed randomized controlled trials are needed to provide high-level evidence to address these important issues. New trials should address aspects of recovery in physical function to fully capture the clinical implications of tourniquet use.

In response to the lack of high-level evidence the primary aim of this randomized trial is to assess the efficacy tourniquet assisted TKA on patient-reported physical function. The secondary aim is to compare post-surgery pain, function in sports and recreation, quality of life, and performance-based physical function. The explorative outcome measures include; use of pain medication, single-fiber muscle damage, and changes in mechanical muscle function. We hypothesize that non-tourniquet assisted TKA will result in superior patient-reported and physical performance-based outcome measures within the first 3-months compared with standard, tourniquet assisted, surgery.

## Methods/Design

### Study design

This trial will comply with CONSORT (Consolidated Standards of Reporting Trials) guidelines [31,32], and has been designed as a prospective, blinded, parallel-group, superiority trial, with balanced randomization [1:1].

### Participants and recruitment procedures

Patients aged 50 years and older with an indication for TKA will be consecutively recruited from the Department of Orthopedic Surgery and Traumatology, Odense University Hospital, Denmark. The criteria for inclusion and exclusion are listed in Table 1.

**Table 1:** Criteria for participants in the study

**Inclusion criteria**	**Exclusion criteria**
Age > 50	Rheumatoid arthritis
	BMI > 35
Able to tolerate spinal anesthesia	Previous knee surgery
	Malignancy
Clinical and radiological knee OA according to the ‘Ahlbäck’ classification system	Muscle disease
	Deep vein thrombosis or other blood coagulation disorders.
	Neuromuscular problems
	Symptomatic bilateral OA, with planned surgery of the contra lateral knee within 1 year
	Decline or unable to participate

OA, Osteoarthritis; BMI, Body Mass Index.

On initial contact, patients will be evaluated by an orthopedic knee surgeon, who will establish the indication for surgery, and verify that all inclusion criteria and none of the exclusion criteria are met (Table 1). Next, the patients will be invited to participate in the trial, and will receive verbal and written information about the trial in accordance with the information sheet approved by the ethics committee. Patients will be given the opportunity to ask questions about the trial and if they wish to participate they will complete a standardized consent form. Patients who do not wish to make a decision regarding participating in the trial during the consultation will be given a stamped addressed envelope and will be contacted by the research nurse no later than a few 4 after the consultation. The research nurse will also ensure that participants have signed the written consent form, are randomized and scheduled for the completion of all baseline measurements. Patients declining to participate in the trial will receive standard surgery (tourniquet-assisted TKA). An independent radiologist will stage the knee osteoarthritis severity using the Ahlbäck classification system [33].

**Figure 1:**
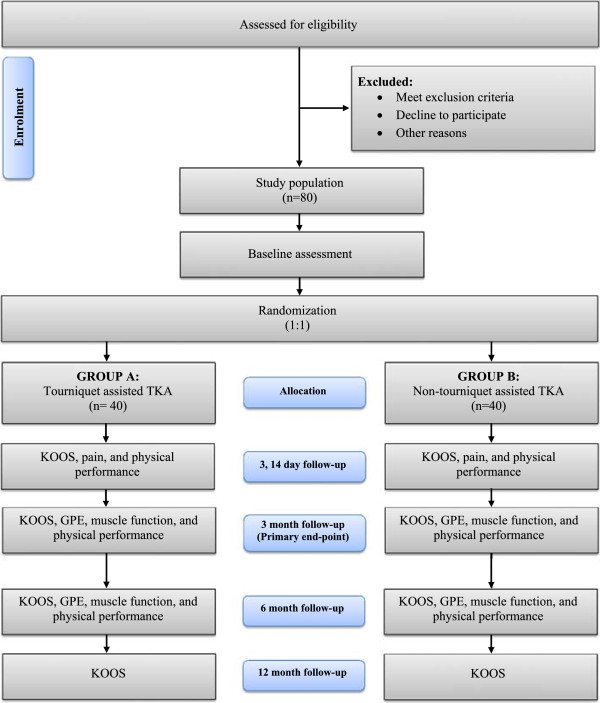
Flow diagram through-out trial.

A compete overview of the recruitment procedures (enrolment, randomization, treatment allocation, follow-up and data analysis) is shown in Figure 1.

### Randomization and allocation concealment

The randomization sequence will be computer-generated using Stata 13.0 (StataCorp, College Station, TX) statistical software with a 1:1 allocation ratio using random block sizes of 10 and 20 patients. Patients will be allocated to one of two treatments by a sequence of letters: A – referring to tourniquet assisted TKA and B – referring to non-tourniquet assisted TKA using sequentially numbered opaque, sealed envelopes. The allocation sequence and preparation of the concealed envelopes will be completed by a third person (JL) not involved in the conduct of the trial.

The allocation sequence will be concealed from the group of surgeons enrolling and assessing participants. Informed consent will be obtained from the participant, in ignorance of the next assignment in the sequence, by a research nurse. To prevent subversion of the allocation sequence, name and date of birth of the participant will be written on the envelope immediately after randomization by the research nurse. Regardless of group allocation, envelopes will first be opened after enrolled patients have completed all the baseline assessments and immediately prior to the surgical intervention.

### Intervention

The entire surgical procedure will be performed under spinal analgesia with a typical duration of 60–90 min and all patients, regardless of length-of-surgery, will remain in the trial population. All patients will undergo surgery using a midline and a medial parapatellar incision through the joint capsule. Patients will receive a cemented total knee arthroplasty (P.F.C.® Sigma®, posterior cruciate retaining knee system, Depuy). Prior to surgery patients receive transexamic acid (Cyclocapron) to decrease bleeding. During surgery, pain management will consist of local infiltration anesthesia (300 mg naropin, 1 mg toradol and 0.5 mg epinephrine periarticularly). The post-surgery pain management will consist of paracetamol (1 g four times/day), contalgin (10 mg two times/day) according to our standard protocol and morphine analogs (10 mg Oxynorm) as required, with the aim of providing sufficient pain relief to facilitate early mobilization. Following discharge patients will receive one-week of anti-thrombosis treatment (fragmin, 5000 IE.sc). A single surgeon trained in surgery with and without the use of a tourniquet will perform both surgical procedures.

#### Group A: the tourniquet assisted TKA group

Patients in the tourniquet group will have surgery performed with an automatic, micro-processor-based pneumatic tourniquet (A.T.S. 2000 tourniquet system, Ref. 60-2000-101-00, Zimmer), patient-dependent size and inflated to 250 mmHg (± 3 mmHg pressure accuracy).

#### Group B: the non-tourniquet assisted TKA group

In the non-tourniquet assisted TKA group the entire operation will be performed without application of a tourniquet.

In-hospital rehabilitation will be standardized for both surgical treatments and consists of the use of crutches, range of motion and stair negotiation exercises all of which are performed under supervision of a physiotherapist. Duration of hospital stay is typically 2 to 3 days and patients are generally discharged with crutches. Following discharge the patients will be provided with a leaflet detailing exercises to continue at home as part of the ongoing rehabilitation process.

### Outcome measures

A single primary outcome measure was chosen to eliminate problems with result interpretation often associated with multiplicity of analysis [34].

#### Primary outcome measure

##### The Knee injury and osteoarthritis outcome score (KOOS-ADL)

KOOS is patient-reported outcome measure with good evidence of reliability, validity and responsiveness in different population with varying pathologies, injury durations and activity levels [35-37]. A 5-point Likert-scale is used and converted into a 100-point scale with zero indicating the worst possible health [35].

#### Secondary outcome measures

Secondary outcomes include two patient-reported and physical performance-based measures:

##### The Knee injury and osteoarthritis outcome score (KOOS)

The remaining 4 subscales for pain, other symptoms, function in sport and recreation and knee-related quality of life.

##### Global perceived effect (GPE) score

Responsiveness is defined as the ability of a patient-reported outcome (PRO) instrument to detect changes over time in the construct to be measured [38]. For evaluating responsiveness, a GPE score, where the patients rate their condition on a 7-point Likert-scale is a recommended responder criteria [39]. The GPE questionnaire has the following seven response options: much better (3), better (2), somewhat better (1), no change (0), somewhat worse (−1), worse (−2) and much worse (−3). A 2-point change from ‘no change’ will be used as cut-off to categorize the response into: i) better, ii) no change, and iii) worse. At the 3-months and 12-months follow-up the patients will be asked to rate possible change in their condition from baseline.

##### Passive knee range of motion (kneeROM)

The number of degrees an examiner is able to move the knee joint through its full range of motion with no active effort from the patient. Mobility is measured in degrees using a standard (30 cm) goniometer [40]. Inter-tester reliability (r = 0.98; ICC = 0.99) and validity (r = 0.97; ICC = 0.98) of goniometer measurements of knee joint motion have been shown [41].

##### The 30-s chair-stand test (30-s CST)

The number of stands, from a seated position with arms folded across the chest, the patient is able to complete within a 30-second time period. CST measures lower-body strength and is an important aspect of fitness in older adults because of its role in common everyday activities. Past studies have shown that chair stand performance correlates well with laboratory measures of lower-body strength [42], and with other indicators of interest such as walking speed, stair climbing ability and balance in older adults [43]. CST has shown good intra- and inter-rater reliability in older adults with OA (ICC = 0.89) [44,45].

##### 20 m self-paced and fast-paced walk test (20mWT)

The time in seconds required to walk a 20 m distance at self- and fast-pace. Walking time have been shown to be reliable (ICC = 0.93 and 0.98, respectively), and valid in reflecting functional performance in knee OA patients [46]. Good agreement and excellent test-retest reliability have been demonstrated in both hip and knee OA patients [44].

##### Timed up-and-go test (TUG)

The time in seconds needed for a patient to rise from an armchair, walk 3 m, turn, and then return to sit on the same chair. The TUG has been shown to be reliable and valid in reflecting performance-based test of mobility [47,48], and activity in daily living evaluated by a patient-reported questionnaire [49].

##### Self-reported pain intensity

Self-reported pain intensity is measured using the continuous (or “analogue”) aspect of the visual analogue scale (VAS), (0 = no pain and 100 = worst possible pain). The VAS scale is reliable and valid in assessing the intensity of musculoskeletal knee pain [50].

#### Explorative outcomes

The explorative part of the trial investigates the changes in a range of muscle mechanical measurements; Isometric knee muscle strength (iMVC) during knee extension and flexion, leg extension power (LEP), rate of force development (RFD), force steadiness [51,52], and surface electromyography (sEMG) supported by single-leg knee bending performance and muscle biopsies.

##### Maximal isometric knee strength

The maximal force (Nm) generated during isometric knee extension and knee flexion. Three consecutive test contractions will be performed, and the peak value will be selected for further analysis [51]. The force-generating capacity is regarded as an essential functional outcome measure, and a prime factor to consider when examining functional limitations following TKA [53]. A comparable isometric testing device has been shown to demonstrate discriminant validity and high test-retest reliability in knee OA patients [54].

##### *Dynamic leg extension power* (LEP)

The maximal power (Watt; force x velocity) generated by the leg extensor muscles during ½-sec unilateral leg extension press in the Nottingham Power Rig. Each leg will be tested separately using 4–6 trial and separated by 30-s rest. Leg extension power has been described in detail [55,56] and tested for reliability in patients with knee OA [55].

##### The rate of force development (RFD_200_)

The change in force generated during the early phase of a muscle contraction (0-200 ms) [52,57]. Several activities, such as descending stairs, fast-paced walking, or prevention of fall are characterized by a limited time to generate force, and the ability to rapidly produce force is an essential functional parameter [2,58].

##### Force steadiness

The ability to produce a given force with a minimal amount of variation, and is routinely used to quantify mechanical and neuromuscular parameters in skeletal muscle [52].

##### Surface Electromyography (sEMG)

The surface myoelectric (sEMG) signals associated with muscle contractions will be collected on lower limb muscles; m. rectus femoris, m. vastus medialis, m. vastus lateralis, m. biceps femoris and semitendinosus following the SENIAM guidelines [59]. EMG will be collected during iMVC and force steadiness muscle contractions. (http://www.SENIAM.org). Good test-retest reliability was reported in healthy subjects [60].

##### 30-s single-leg knee bending (30s-KneeBend)

The maximum number of bends completed within a 30 second time period. 30s-KneeBend measures the ability to execute fast stretch shortening cycles over the knee joint. The test is valid in discriminating between the symptomatic and the non-symptomatic leg in meniscectomized patients [61] and moderate agreement and good test-retest reliability in patients with hip and knee OA has been reported [44].

##### Single-fiber muscle damage

Muscle biopsy samples (2 × 100 mg) will be acquired during surgery in a subgroup of the patients (2 × 10). One sample will be collected prior to applying the tourniquet, and a second sample will be collected prior to the removal of the tourniquet. The muscle sample will be taken in every 4^th^ patient (e.g. patient no. 4, 8 and 12 etc.) Since the randomization sequence is concealed the unblinded surgeon will be responsible for selecting the next patient in line to ensure an equal distribution of samples drawn from each treatment group. The muscle biopsies will be performed through a small surgical incision into m. quadriceps femoris, and directly beneath the area where the tourniquet was applied. The muscle tissue samples will be frozen in liquid nitrogen, and saved for later analysis (single-fiber muscle isometric mechanical damage/function; force, force/cross sectional area, Ca^++^-sensitivity, and fiber stiffness).

### Adverse events

#### Surgery related data

A medical record audit will be performed at 3 month post-surgery where, surgery-time, intraoperative blood loss, wound infection, deep-vein thrombosis, pulmonary embolism, time-to-ambulation, prosthetic loosening, revisions will be reviewed and summarized. Also, the degree of knee varus/valgus before and after surgery will be evaluated.

#### Self-reported use of pain medication

The patient’s use of pain medication will be registered using a diary.

### Follow-up period

Assessments will be performed pre-surgery, 3, 14 days and 3, 6, 12-months after surgery with 3-months as the primary end-point. A summary of the outcome measures and time-points can be found in Table 2.

**Table 2:** Summary of outcome measures to be collected in the trial with time-points

**Primary outcome**	**Data collection instrument (unit)**	**Collection time-point**		
		**Baseline**	**Post (days)**	**Post (months)**
Function in daily living	KOOS	Pre	14	3,6,12
**Secondary outcome**				
KOOS - 4 subscales				
Pain	KOOS	Pre	14	3,6,12
Symptoms	KOOS	Pre	14	3,6,12
Sport and recreation	KOOS	Pre	14	3,6,12
Quality of life	KOOS	Pre	14	3,6,12
Questionnaire				
Global perceived effect	GPE			3,6,12
Bilateral physical Performance				
Knee range of motion (KneeROM)	Goniometer (°)	Pre	3,14	3,6
30-s chair-stand test (30s CRT)	(Number)	Pre	3,14	3,6
20-m self- and fast-paced walk test (20mWT)	Stopwatch (s)	Pre	3,14	3,6
Timed up-and-go test (TUG)	Stopwatch (s)	Pre	3,14	3,6
**Explorative outcomes**				
Mechanical muscle function				
Maximal isometric knee strength (iMVC)	Strain-gauge (Nm)	Pre		3,6
Dynamic leg extension power	(W)	Pre		3,6
Rate of force development (RFD)	(Nm/sec)	Pre		3,6
Surface electromyography (EMG)	Myon (mV)	Pre		3,6
Force steadiness	(Nm)	Pre		3,6
Unilateral physical performance				
30-s single-leg knee bending (30s-KneeBend)	(Number)	Pre		3,6
Pain				
Self-reported use of pain medication	(Quantity)	Pre	1-14	
Self-reported pain (VAS)	VAS	Pre	1-14	
Biopsies				
Muscle biopsy		Prior to applying and removal of the tourniquet		

### Blinding

Blinding to treatment allocation (surgeons) is not possible due to the nature of the surgical intervention. However, the unblinded surgeon will not be involved in any care or outcome assessments. Multiple strategies will be used to maximize patients blinding in this trial. Firstly, patients will not be informed about their surgical treatment, but swelling may reveal if tourniquet was used. Second, all patients will be requested not to disclose details about their treatment to the data collectors, should they become aware of their allocation. Third, the tourniquet will first be applied when patients are under anesthesia and lightly sedated. Fourth, postsurgical care regime will be standardized for both treatment groups. Finally, identical setting, positioning of patients, masking by drape and post-surgical sham dressing will be applied.

Several strategies will be used to blind the data collector and principal investigator. The data collector responsible for baseline and follow-up assessments will be an independent person. All patient-reported outcome measures will be entered into a database using optical character recognition (OCR) scanning software and subsequently identified by trial numbers only. The principal investigator and data analyst (RLJ) will be blinded to the allocation sequence as data will be analyzed using recoded trial numbers only. The recoding of the trial identification numbers will be performed by an independent person (JL).

### Statistical analysis

The primary statistical analysis will be performed on KOOS-ADL 3-month following surgery. To evaluate the treatment effect (mean difference in KOOS scores) we will employ a random or fixed effects linear regression model with point estimates [62] and both crude and adjusted results will be reported. The regression model includes the interaction between treatment and elapsed time, adjusted for pre-surgery values and assumes data is missing completely at random (MCAR) [62]. Model assumptions will be checked by residual plots. The secondary statistical analysis will include the same approach as described above for all the secondary outcomes. All analyses will be performed on the basis of intention-to-treat (ITT) [63]. However, subsequent per-protocol analysis may be necessary in the event of a substantial number of patients (> 15%) being lost during follow-up. All data will be checked for Gaussian distribution and parametric statistics will be used were appropriate, otherwise non-parametric statistics will be applied. Finally, the number needed to treat (NNT) for a positive effect of treatment (>10 points on KOOS-ADL) will be analyzed. All statistical analyses are blinded and will be performed using Stata software (StataCorp, Texas, USA).

### Sample size

Sample size estimation was performed upon the primary outcome KOOS-ADL, using one pre-surgery and 2 follow-up assessments and an estimated correlation between follow-up measurements of 0.5. Based upon data from a non-randomized, but controlled study from Sweden, a 10-point change in KOOS-ADL (SD of 18.5 pre-surgery and 10.4 post-surgery) has been selected as clinical important difference at the 3-months end-point. A sample size of n = 36 is needed in each group (α = 0.05, β = 0.80)). To account for possible drop-outs n = 40 will be included in each group.

### Timeline and ethics

Recruitment of patients is scheduled to begin in spring 2014 and will last for 1 year. All patients are expected to have completed the trial by spring 2016. The omission of tourniquet use is expected to only marginally increase surgery time (~ 5 min.), and the increased surgery time is unlikely to increase the risk of complications. In contrast, total theatre time is expected to remain unchanged due to the time saved when application of the tourniquet is omitted. No difference, or a slight non-critical increase, in total blood loss in the non-tourniquet group is expected [15]. The trial complies with the Declaration of Helsinki and the trial has been approved by the Regional Ethics Committee of Southern Denmark (S-20110084).

## Discussion

This clinical trial will evaluate the efficacy of tourniquet assisted TKA on patient-reported and performance-based physical performance As a prospective randomized controlled trial the results of this study will provide a high level of evidence on the clinical implications of using a tourniquet during TKA. The primary end point, at 3-month, is a sufficiently long timeframe for the relevant clinical improvements between groups to show and yet short enough to assume that patients can recall their baseline condition. As part of the design, a single senior surgeon will perform the surgical procedures in both groups. The experience of the surgeon in both tourniquet-assisted and non-tourniquet TKA and use of random block size randomization will eliminate bias due to learning. Additionally use of a single surgeon will ensure we are comparing the TKA procedure rather than the skill of different surgeons.

The key aim of TKA in patients with OA is to relieve pain and improve function, both of which are patient reported outcome (PROs) measures reflected in the design of this trial and recommended outcome measures by the Osteoarthritis Research Society International (OARSI) and Outcome Measures in Rheumatology and Clinical Trials (OMERACT) group [39]. Ideally, changes in physical function following TKA should be evaluated using a combination of patient-reported outcomes (PROs) and performance-based outcome measures [39,45] as they are complementary rather than competing [64,65] and capture different constructs of physical function [66,67]. In addition, responsiveness of a PROs ability to detect when patients are undergoing relevant clinical changes can be assessed using a global response evaluation (GPE), and a GPE questionnaire is a recommended part of the evaluation of a treatment in clinical trials [38,39]. To accommodate these recommendations, the patients in the present trial will be asked to rate their current knee condition pre-surgery (baseline), and at each follow-up (KOOS) and also rate changes in their condition since baseline (GPE). These two patient-reported outcome measures (KOOS and GPE) will be supported by assessor-observed performance-based tests of physical function, hence evaluating, what ‘patients can do’ rather than what they ‘perceive they can do’ [67].

According to WHOs classification of Functioning, Disability and Health (ICF) physical function can be classified as the ability to “perform daily activities” [68]. Hence, to fully capture physical function in patients following joint replacement, performance-based testing should represent a variety of physical function domains (activity themes) representing the following five daily activities; *walking, sit-to-stand, ambulatory transitions, stair negotiation, and endurance*[45]. The current trial uses the 2×20 m fast-paced walk test, the 30-s chair-stand test, and the timed up-and-go test evaluate; walking, sit-to-stand, and ambulatory transition activity domains, repetitively. Despite the importance of also evaluating *stair negotiation activity,* no specific test is planned in the trial for two reasons. First, no recommendations about specific performance-based tests of physical function, with good measurement-property evidence (reliability, validity, responsiveness, and interpretability), is currently available. Second, the feasibility of stair negotiation tests is largely dependent on the environmental setting making them unsuitable for clinical trials at present [45]. Likewise, no test for evaluating *endurance activity* has been planned for this trial due to the practical feasibilities issues associated with the six-minute walk test, e.g. patients are unable to walk for six minutes and exhausted following surgery. Further, endurance activity evaluation may be compromised in the early post-surgery phase since pain and swelling, rather than endurance, could limit patients’ performance. Consequently the current trial evaluates three primary activity themes using a core set of performance-based tests recommended by OARSI [45], and from this core set of tests efficient comparisons of treatment outcomes across published literature can be made.

Evaluating the domain of pain or physical function in patients following joint replacement surgery is important [39]. However, the decision to nominate physical function as primary outcome was due to two reasons. The KOOS questionnaire evaluates physical function in a variety of daily activities, and has an extensive subscale (KOOS-ADL, 17 items) devoted to evaluate physical function compared to the pain-subscale (KOOS-PS, 10 items). Additionally, WOMAC and KOOS are the most common PROs used to capture patient relevant information, relating to the impact of interventions in clinical research [69]. WOMAC scores can subsequently be calculated based on data collected using the KOOS questionnaire, thus making efficient comparisons across published literature possible.

Essential explorative outcome measures are also included in the present trial to study muscle mechanics and potential microscopic muscle damage. Studying muscle mechanics is clinical relevant in understanding body structure [51,70] and the recovery of muscle strength and power are likely to be compromised in tourniquet assisted TKA patients. The theoretical disadvantages of tourniquet application on muscle mechanics is supported by one randomized trial [7] and two non-controlled studies in which reduced muscle function and neurological impairment have been reported [29,71]. However, such findings should be confirmed in high-quality trials using randomized designs.

A number of other factors may also influence the effects of tourniquets [15]. It’s been suggested that the tourniquet position may be of clinical importance [72] but also cuff width, cuff pressure, time of cuff deflation (cementation of the prosthesis, would closure or application of dressing) must be considered factors that may affect outcome, and should be recorded [15]. Other factors include use of thromboprophylaxis, girth/circumference of thigh, and patient blood pressure. Data on these variables will be collected to insure uniform presentation of data in peer-reviewed papers [9,15].

## Conclusion

We have designed a randomized clinical trial with the main purpose of investigating the efficacy of tourniquet assisted TKA on patient-reported physical function, an outcome which will be supported by a range of performance-based secondary outcome measures. This part of the trial will provide results with high-level evidence and may help to determine whether tourniquet should be used in future TKA procedures for those with severe osteoarthritis of the knee. The explorative part of the trial could also provide further understanding of the underlying neurological and muscle mechanical impairments associated with use of tourniquet during TKA surgery. The results will be submitted to a peer-reviewed international journal for publication irrespective of the outcome in accordance with the CONSORT guidelines for reporting of clinical trials.

## Abbreviations

ADL: Activities of daily living; GPE: Global perceived effect; KOOS: The knee disability and osteoarthritis outcome score; iMVC: Isometric maximal voluntary contraction; QoL: Quality of life; TKA: Total knee arthroplasty; VAS: Visual analog scale; LEP: Leg extension power.

## Competing interests

The authors declare that they have no competing interests.

## Authors’ contributions

RLJ will manage the coordination of the trial. RLJ and CJ were responsible for drafting this paper. AHL, SO and CJ conceived the project while SO/CJ/RLJ procured the project funding. CE will be responsible for planning and execution of the knee arthroplasty. All authors provided intellectual input on the design, feedback on drafts of this protocol, and approved the final manuscript.

## Pre-publication history

The pre-publication history for this paper can be accessed here:

http://www.biomedcentral.com/1471-2474/15/110/prepub
